# Evaluation of antiaggregatory activity of flavonoid aglycone series

**DOI:** 10.1186/1475-2891-10-73

**Published:** 2011-07-11

**Authors:** Mirza Bojić, Željko Debeljak, Maja Tomičić, Marica Medić-Šarić, Siniša Tomić

**Affiliations:** 1 University of Zagreb, Faculty of Pharmacy and Biochemistry, Department of Medicinal Chemistry, A. Kovačića 1, 10000 Zagreb, Croatia; 2 Department of Clinical Laboratory Diagnostics, CHC Osijek, J. Huttlera 4, 31000 Osijek, Croatia; 3 Croatian Institute of Transfusion Medicine, Department of Platelet and Leukocyte Immunology, Petrova 3, 10000 Zagreb, Croatia; 4 Agency for Medicinal Products and Medical Devices of Croatia, Ksaverska cesta 4, Zagreb, Croatia

## Abstract

**Background:**

Among natural compounds, present in every day diet, flavonoids have shown beneficial effect in prevention of cardiovascular diseases that can be attributed, at least partially to the described antiaggregatory activity i.e. antiplatelet effects of flavonoids. Due to the ever increasing pharmacological interest in antiplatelet agents a systematic experimental evaluation of large flavonoid series is needed.

**Methods:**

A set of thirty flavonoid aglycones has been selected for the evaluation. All measurements of aggregation were done under standardized and firmly controlled *in vitro *conditions. The whole blood samples, multiple platelet functional analyzer and adenosine diphosphate (ADP) as a weak agonist of aggregation were selected for this purpose.

**Results:**

The results were expressed as minimal concentration of flavonoid that can significantly lower the platelet aggregation compared to the corresponding untreated sample (minimal antiaggregatory concentration - *MINaAC*). All analyzed flavonoids exhibited antiaggregatory activity *MINaAC *ranging from 0.119 μM to 122 μM, while the most potent representatives were 3,6-dihydroxyflavone (0.119 μM) and syringetin (0.119 μM).

**Conclusions:**

Measurable antiplatelet activity established at submicromolar flavonoid concentrations suggests that even a dietary consumption of some flavonoids can make an impact on *in vivo *aggregation of platelets. These findings also point out a therapeutical potential of some flavonoids.

## Background

**Figure 1:**
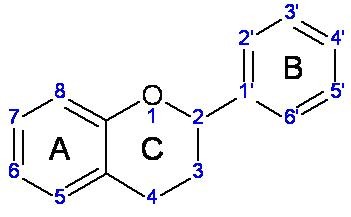
**Basic structure of flavonoids**. Flavonoids are divided into classes based on the structure of ring C. Basic structure corresponds to flavan which are named flavanols (catechins) if hydroxylated at position C3. Flavanones have keto group on position C4. If the double bond C2 = C3 is present in structure flavanols and flavanones are named flavones and flavanonols, respectively. Isoflavonoids have B-ring at the position C3.

In the developed countries most of the older population is affected by cardiovascular diseases. Platelets are involved in haemostasis, thrombosis and inflammatory processes, hence as a consequence of that physiological role heart stroke and cerebrovascular insult can occur. Most commonly used drug in prevention of mentioned diseases is acetylsalicylic acid while clopidogrel represents another therapeutic option. Neither of these drugs is free of side effects, thus the search for new and safer drug from this group continues [1]. From the natural compounds, present in every day diet, polyphenols, mainly flavonoids (Figure 1), have shown beneficial effect in prevention of cardiovascular diseases [2-7]. Flavonoids naturally occur in a free form (aglycones) or bound to a sugar moiety *via *hydroxyl groups (glycosides).

Flavonoid antiplatelet activity can be attributed to the increased production of prostacyclin by endothelian cells. Prostacyclin decreases aggregation *via *synthesis of cAMP - increased concentration of cAMP inhibits the expression of platelet GPIIb/IIIa receptors [6]. *In vitro *inhibition of cyclooxygenase, lipooxygenase, thyrosine kinase, phosphodiesterase or phospholipase by flavonoids has also been documented and connected to their antiplatelet activity [8-12]. Although different possible mechanisms have been analyzed a unique mechanism of antiaggregatory activity of flavonoids has not been undoubtedly proven yet.

There are also doubts about antiaggregatory effectiveness *in vivo*, due to high concentrations of flavonoids that have been used in experiments *in vitro *(10 - 1000 μM) that can not be reached *in vivo *after oral intake (0.6 μM) [13].

Most of the research on antiaggregatory effect of flavonoids has been done using Born spectrophotometric aggregometry. A major disadvantage of this method is usage of platelets rich plasma (PRP) instead of whole blood. Furthermore, most authors tested a range or even a single concentration of flavonoids, and often the series of tested flavonoids were small, thus limiting the overall interpretation of the results [9,14-18]. Finally, influence of biological variability has not been evaluated.

In this paper our objective was to analyse antiaggregatory effect of a relatively large set consisting of 30 flavonoid aglycons. Instead of Born method impedance aggregometry has been chosen as it enables usage of whole blood. This method reduces problems related to lack of standardization of PRP preparation and provides insight to possible interactions of flavonoids with blood components other than platelets. Biological triplicates of all experiments have been made on blood samples taken from three different blood donors. Along with the statistical evaluation of difference between treated and untreated samples this approach clearly minimizes chance effects caused by large biological variability.

## Methods

### Materials

A set of thirty flavonoids has been tested. Structures and the names of the suppliers are stated in the Table 1, Table 2, Table 3, Table 4 and Table 5. Clopidogrel, an ADP-receptor antagonist, was used as a positive control. This substance was a kind gift of HALMED, Croatia. All standards solutions were prepared by dissolving and semi-dissolution (1/2^n^) in dimethyl sulfoxide (DMSO, Sigma-Aldrich, Switzerland) in the concentration range of 500 mM to 30 nM depending of the flavonoid analyzed. Final concentration of DMSO in all experiments was 3%.

**Table 1:** Antiaggregatory activity of flavanons

Flavanons	Structure	*MINaAC*/μM	*p*
Hesperetin^a^		1.907	0.035

Homoeriodictyol^b^		7.629	0.003

Isosakuranetin^b^		0.954	0.037

Pinocembrin^c^		15.259	0.010

Pinocembrin-7-methylether^c^		0.954	0.025

Minimal antiaggregatory concentration of flavanons expressed in μM with statistical significance (*p*).

Flavonoids purchased from ^a^Sigma-Aldrich, Switzerland, ^b^BioChemika, Switzerland, ^c^Extrasynthese, France.

**Table 2:** Antiaggregatory activity of flavones

Flavones	Structure	*MINaAC*/μM	*p*
6-hydroxyflavone^a^		0.954	0.030

7-hydroxyflavone^a^		15.259	0.038

Acacetin^b^		3.815	0.013

Apigenin^c^		3.815	0.037

Chrysin^c^		3.815	0.016

Chrysin dimethylether^d^		1.907	0.025

Diosmetin^d^		7.629	0.021

Flavone^c^		3.815	0.037

Luteolin^d^		7.629	0.029

Tangeretin^b^		30.518	0.029

Tectochrysin^d^		0.954	0.013

Minimal antiaggregatory concentration of flavones expressed in μM with statistical significance (*p*).

Flavonoids purchased from ^a^ChromaDex, USA, ^b^BioChemika, Switzerland, ^c^Fluka, Germany, ^d^Extrasynthese, France.

**Table 3:** Antiaggregatory activity of flavanonols

Flavanonols	Structure	*MINaAC*/μM	*p*
3-hydroxyflavone^a^		1.907	0.019

3,6-dihydroxyflavone^a^		0.119	0.005

3,7-dihydroxyflavone^a^		1.907	0.001

Fisetin^b^		122.070	0.003

Galangin^c^		122.070	0.008

Isorhamnetin^d^		7.629	0.029

Quercetin^e,*^		15.259	0.047

Rhamnetin^d^		0.954	0.041

Syringetin^d^		0.119	0.013

Minimal antiaggregatory concentration of flavanonols expressed in μM with statistical significance (*p*).

Flavonoids purchased from ^a^ChromaDex, USA, ^b^Extrasynthese, France, ^c^Sigma-Aldrich, Switzerland, ^d^BioChemika, Switzerland, ^e^Fluka, Germany; *in the form of quercetin dihydrate.

**Table 4:** Antiaggregatory activity of isoflavones

Isoflavones	Structure	*MINaAC*/μM	*p*
Daidzein^a^		15.259	0.048

Formononetin^a^		7.629	0.043

Genistein^a^		30.518	0.013

Prunetin^a^		7.629	0.033

Minimal antiaggregatory concentration of isoflavones expressed in μM with statistical significance (*p*).

Flavonoids purchased from ^a^Extrasynthese, France.

**Table 5:** Antiaggregatory activity of positive control and catechin

Substance	Structure	*MINaAC*/μM	*p*
Epicatechin^a^		1.907	0.030

Clopidogrel		0.019	0.029

Minimal antiaggregatory concentration of positive control and catechin expressed in μM with statistical significance (*p*).

Flavonoid purchased from ^a^Sigma-Aldrich, Switzerland.

ADP was obtained from Dynabyte, Germany, and saline-CaCl_2 _(0.003 M CaCl_2 _in 0.9% NaCl) from Croatian Institute of Transfusion Medicine, Croatia.

Freshly taken citrated blood (final citrate concentration 0.129 mol/L) was used for the measurement of aggregation each time from three different healthy volunteers per each flavonoid sample. This work was approved by Ethical comities of Croatian Institute of Transfusion Medicine and Faculty of Pharmacy and Biochemistry, University of Zagreb. A total number of 100 volunteers participated in this research. All volunteers gave informed written consent.

### Experimental procedure

Platelet aggregation was analyzed by Multiplate^® ^analyzer (Dynabyte, Germany). Generic procedure was used: 300 μL of blood was incubated for 6 minutes with 20 μL flavonoid solution and 300 μL of saline-CaCl_2 _preheated at 37°C. For negative control (untreated sample) 20 μL of solvent - DMSO was used (final concentration 3%). Aggregation cascade was induced by adding 20 μL of adenosine diphosphate reagent (ADPtest; final concentration of ADP 6.5 μM). Aggregation was measured for 6 minutes and expressed as area under curve in arbitrary units (AU).

### Data analysis

**Figure 2:**
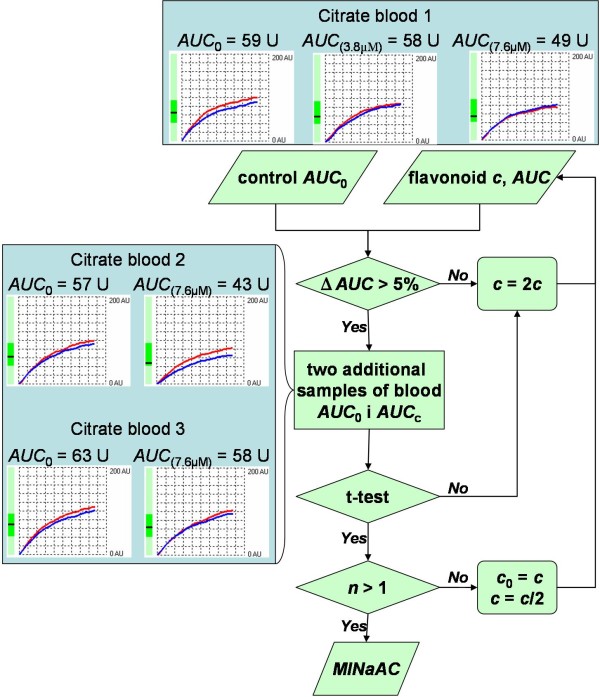
**Design of the experiment**. Aggregation of untreated sample (without adding the flavonoid, *AUC*_0_) and the sample of flavonoid (*AUC*) are measured. If the difference of the aggregations is greater than 5% the measurement of additional two citrated bloods is performed. Otherwise, double concentration of flavonoid is taken and the first step is repeated. If the flavonoid in the analysed concentration shows statistically lower aggregation compared to the untreated sample (t-test) the analysed concentration is equal to minimal antiaggregatory concentration (*MINaAC*). If not analysis is performed from the beginning with double the concentration of flavonoid. This presumes that the analyzed concentration is not the first analysed (*n *> 1), else, the analysis is performed from beginning using the half of the concentration of flavonoid. Example of prunetin is given. Aggregation of analysed concentration of prunetin: 3.8 μM is not greater than aggregation of the untreated sample (58 U *vs*. 59 U). Thus additional measurement with double concentration (7.6 μM) of prunetin is performed. As the difference of aggregation is greater than 5% (Δ*AUC *= 17%) measurements on two additional citrated blood samples were performed. The concentration obtained is statistically different compared to the untreated sample (*p *= 0.033) thus minimal antiaggregatory concentration of prunetin is equal 7.6 μM. Note that red and blue lines in charts indicate two parallel measurements of the same sample. If the deviation between these two measurements is greater than 10%, quality control flag appears and measurements should be repeated.

The results of antiaggregatory effect of flavonoids were expressed as minimal antiaggregatory concentration (*MINaAC*) that presents the lowest concentration of flavonoid which can cause statistically significant reduction of aggregation when compared to the untreated sample. Procedure for determination of *MINaAC *is illustrated on Figure 2. Statistical analysis was performed using paired Student's t-test within R v2.8.1 environment (Austria). Normal distribution of aggregation on ten healthy volunteers was checked using Shapiro-Wilk test (*p *= 0.501) justifying the use of t-test.

## Results and Discussion

All analyzed flavonoids exhibited antiaggregatory activity with *MINaAC *ranging from 119 nM to 122 μM.

Pinocembrine-7-methylether has lower *MINaAC *than pinocembrin, thus methylated derivates of flavanons have greater antiaggregatory effect. Most potent flavanones are p-O-methylated derivates at B-ring (Table 1).

Monohydroxylated flavones are most potent if substituted at the position 6 of the A-ring (6-hydroxyflavone, Table 2). However, most naturally occurring flavonoids are hydroxylated at position 7 (7-hydroxyflavone), thus having lower antiaggregatory effect. Increase in number of hydroxyl groups does not influence antiaggregatory effect (flavone, chrysin, apigenin). Methylation increases antiaggregatory effect, the same as it does in case of flavones (tectochrysin > chrysin). If the flavone has more than four substituents the activity decreases, even if methylated (diosmetin, luteolin, tangeretin).

Generally hydoxylated flavanons have greater antiaggregatory effect than flavones (pinocembrin > chrysin), opposite to methylated derivates (hesperetin > diosmetin, isosakuranetin > acacetin, pinocembrin-7-methylether = tectochrysin).

Comparing monohydroxylated flavanonols to monohydroxylated flavones antiaggregatory effect is higher (3,6-dihydroxyflavone > 6-hydroxyflavone, 3,7-dihydroxyflavone > 7-hydroxyflavone). As it is the case with flavones, increase in number of substituents (four and more) decreases, while methylation increases antiaggregatory effect (syringetin > rhamnetin > quercetin, Table 3).

Isoflavonoids are less potent antiaggregatory agents, but the same as for flavonoids applies: increase in hydroxylation decreases while methylation increases antiaggregatory effect (Table 4). This is probably due to greater volume and higher lipophilicity of the methyl radical (*V *= 37.15, *π *= -0.09) compared to hydroxyl group (*V *= 11.79, *π *= -0.74). Higher lipophilicity can lead to significant interactions with the platelet's membrane by increasing rigidity. The membrane is stabilized and the appearance of the receptors - integrins e.g. GPIIa/IIIb at the platelet surface is limited [19-21].

Epicatechin, present in wine, was the only catechin analyzed (Table 5). It has greater antiaggregatory effect compared to flavone and flavanonol parallels, namely luteolin and quercetin.

Based on these observations, structure activity relationship between flavonoids (Figure 1) and antiaggregatory activity reveals that:

- double bond at the position C2-C3 increases activity for hydroxylated derivates, but decreases activity for methylated derivates at the ring A and B,

- hydroxyl group at the position C3 increases antiaggregatory activity,

- transfer of B ring from C2 to C3 decreases activity (isoflavonoids),

- absence of carbonyl group at the position C4 increases activity,

- most potent flavonoids are substituted at the position C6 of the ring A,

- O-methylation of the rings A and B increases activity,

- if the rings A and B have 4 and more radicals activity decreases.

The results of antiaggregatory effect of flavonoids were compared to clopidogrel which acts as antagonist of ADP and serves as positive control in the experiment (Table 5). Although results for clopidogrel are comparable to results of the most potent flavonoids 3,6-dihydroxyflavone and syringetin, this should be interpreted with caution. Clopidogrel is a pro-drug whose metabolic activation to some extent can occur *in vitro *as main enzyme responsible for activation CYP2C19 is present in different tissues including the blood [22].

Dell'Agli *et al*. reported that inhibitory effect on platelet aggregation (induced by thrombin) of 10 μM concentration of individual compound followed the order: luteolin, quercetin and apigenin, the last being inactive [9]. In our study *MINaAC *followed the other arrangement: apigenin (3.815 μM), luteolin (7.629 μM), quercetin (15.259 μM), using ADP to induce aggregation.

Comparing the sets of flavonoids analyzed in our research to the work of Navarro-Nuñez *et al*. where antigreggatory effect was expressed as percentage of inhibition of SQ 29548 binding to thromboxane A_2 _(TxA_2_) receptor, apigenin was the most potent antagonist of aggregation [23]. Thus, it achieves antiaggregatory effect both through antagonism of thromboxane A_2 _receptors as well as ADP receptors. However, genistein was next potent antagonist of TxA_2 _receptors, contrary to our research where isoflavonoids showed lower antagonism to ADP receptors compared to other classes of flavonoids. Hesperetin has *MINaAC *of 1.907 μM, ten to thirty times lower than reported by Jin *et al*., as expected due to usage of strong agonists of aggregation (collagen, arachidonic acid) [17].

The daily consumption of flavonoids is reported to be *m *= 23 mg per day [24]. If we take into account that absorption of flavonoid aglycone is *f *= 24% (as reported for quercetin [25]) of the consumed dose it would mean that concentration which can be achieved with normal diet is:

where *M *represents average molar mass of flavonoid being 300 g/mol and *V *total blood volume of 5 L.

The most potent flavonoids cause minimal antiaggregatory effect in even lower concentrations: 3,6-dihyroxyflavone, syringetin (*MINaAC *= 0.119 μM), 6-hyroxyflavone, pinocembrin-7-methylether, tectochrysin, rhamnetin and isosakuranetin (*MINaAC *= 0.954 μM). These levels of flavonoids in serum have been experimentally confirmed for the most commonly consumed flavonoids: hesperetin and naringenin from orange juice, and epi/catechins from coco and green tea - ranging from 0.01 to 1.1 μM for individual flavonoid [26-29]. This is contrary to findings of Janssen *et al*. that flavonoids are active in concentrations that can not be achieved *in vivo *[13].

## Conclusions

This work provides insight in the antiaggregatory activity of a relatively large set of flavonoid aglycons measured by standardized impendance aggregometry in whole blood against ADP as an inductor. SAR of flavonoids shows that increase in the number of hydroxyl groups at the rings A and B, decreases activity. On the other hand, if O-methyl groups are introduced activity increases. This indicates that the size/volume and lipophilicity of the radical is important factor, which should be confirmed in further QSAR prediction studies.

The results obtained suggest that even a daily consumption of flavonoids can effect aggregation of platelets. As flavonoids are ubiquitous substances in plants, this work can serve as source of information for further assessment of food influence on antiaggregation/anticoagulation treatment.

## Competing interests

The authors declare that they have no competing interests.

## Authors' contributions

MMŠ and ŽD contributed to experimental design. MB and MT carried out the experiments. MB, ŽD, MMŠ and ST contributed to the analysis of the data. MB was principally responsible for writing the paper with assistance from MMŠ and ŽD. All authors read and approved the final manuscript.
